# Immobility and risk of death in patients on invasive mechanical ventilation

**DOI:** 10.1186/2197-425X-3-S1-A163

**Published:** 2015-10-01

**Authors:** D García Huertas, F Manzano Manzano, MM Jiménez Quintana, C Rodríguez Mejías, F Santiago Ruiz, MR Mañas Vera, M Colmenero Ruiz

**Affiliations:** Hospital Virgen de las Nieves, Granada, Spain; Hospital Dr. José Molina Orosa, Lanzarote, Spain; Hospital Universitario San Cecilio, Granada, Spain

## Introduction

The immobility is long recognized as a cause of complications in critical patients, including muscle atrophy, pressure ulcers, atelectasis and ocassionally, mortality. Therefore, the role of mobilizing in critical patients is debated.

## Objective

To study the relationship between immobility and hospital mortality on patients requiring mechanical ventilation (MV) during Intensive Care Unit (ICU) stay.

## Method

A cohort research carried out in a medical-surgical ICU at a third level hospital for 2 years (February 2009-January 2011) on patients who require MV≥ 24 hrs. Data source: clinical trial PUPPAS (1). Primary end point was hospital mortality. The main outcome measure was the implementation of posture changes during the stay in ICU (the percentage of posture changes made with regard to the total of posture changes scheduled). The posture changes include the next sequence of turning schedules every 2-4 hours: supine position, right side, prone position, left side. Secondary predictors of mortality were age, gender, weight, cancer, surgery, MV duration, APACHE II score, duration of anaemia (days), reintubation (%), SOFA 3 score, albuminemia level and incidence of airway obstruction. Statistical research: descriptive, t-student, chi-square and Cox regression model.

## Results

329 patients were included, of 61 ± 25 years, 33% of them were women. APACHE II score was 23 ± 4 and the MV duration was 12,7 ± 18 days. The implementation of posture changes for patients was 61%. Hospital mortality was 63,5 ± 38,6 %. In this multivariable model, the hospital mortality hazard ratio (HR) in relation with the percentage of posture changes implemented was 0,97 (IC 95% 0,96-0,98; p = 0,001). Other variables included on the model were APACHE II (HR 1,076, IC 95% 1,040-1,114; p < 0,001), age (HR 1,024, IC 95% 1,008-1,041; p = 0,004), surgery (HR 0,35 IC 95% 0,22-0,54, p = 0,001), SOFA score on the 3th day (HR 1,097, IC 95% 1,031-1,167; p = 0,003), duration of anaemia (HR 0,95, IC 95% 0,92-0,99; p = 0,02).

## Conclusions

Patients immobility is associated to a risk of death in patients under mechanical ventilation in ICU.
